# Supplementary material for: GABA-A Alpha 2/3 but Not Alpha 1 Receptor Subunit Ligand Inhibits Harmaline and Pimozide-Induced Tremor in Rats
Source: Biomolecules. 2023 Jan 18;13(2):197. doi: 10.3390/biom13020197 (PMC9953228; doi:10.3390/biom13020197)
Supplement: Supplementary file 1 [file biomolecules-13-00197-s001.zip › biomolecules-2097367-supplementary.pdf]

Supplementary Results

GABA-A alpha 2/3 but not alpha 1 receptor subunit ligand inhibits harmaline and pimozide-induced tremor in rats

Barbara Kosmowska1, Martyna Paleczna1, Dominika Biała1, Justyna Kadłuczka1, Jadwiga Wardas1, Jeffrey M. Witkin2,3, James M. Cook2,3, Dishary Sharmin2, Monika Marcinkowska4, Katarzyna Z. Kuter1*

1 Department of Neuropsychopharmacology, Maj Institute of Pharmacology, Polish Academy of Sciences, 12 Smetna St., 31-343 Krakow, Poland

2 Department of Chemistry and Biochemistry, University of Wisconsin-Milwaukee, Milwaukee, WI, USA

3 RespireRx Pharmaceuticals Inc, Glen Rock, NJ, USA

4 Department of Pharmaceutical Chemistry, Jagiellonian University, Medical College, 9 Medyczna St., 30-688 Krakow, Poland

* Correspondence: kuter@if-pan.krakow.pl; Tel.: +48 12 6623226

Figure S1. Tremolous Jaw Movements and Catalepsy induced in Wistar rat by 7 doses of Pimozide.

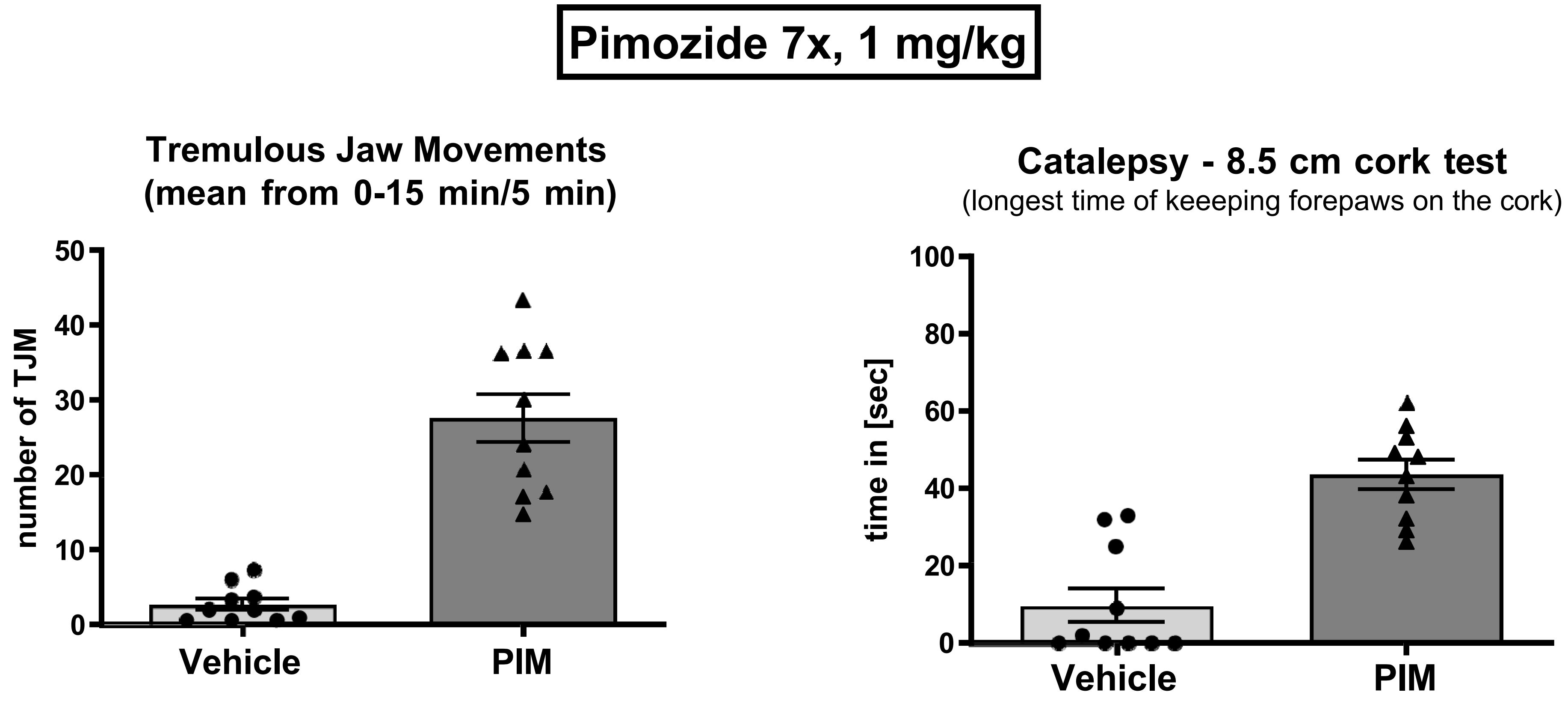

Barbara Kosmowska and Jadwiga Wardas

Figure S2. Tremolous Jaw Movements and Catalepsy induced in Wistar rat by 1 dose of Tetrabenazine.

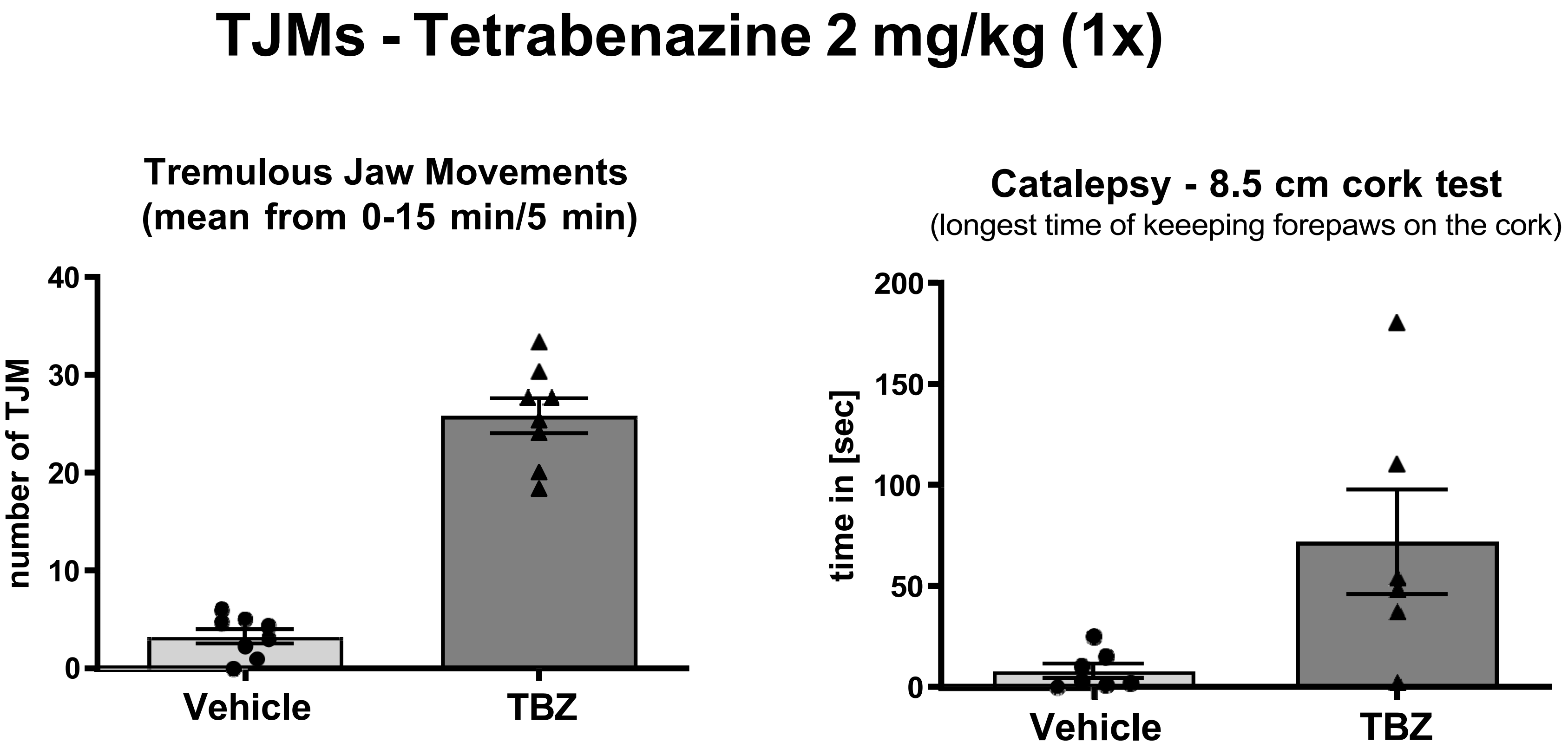

Barbara Kosmowska and Jadwiga Wardas

Figure S3. Walking distance affected by Pimozide or Tetrabenazine, measured in Force Plate Actimeters (BASi, West Lafayette, IN, USA)

Walking distance

PIMOZIDE 1 mg/kg, 7x

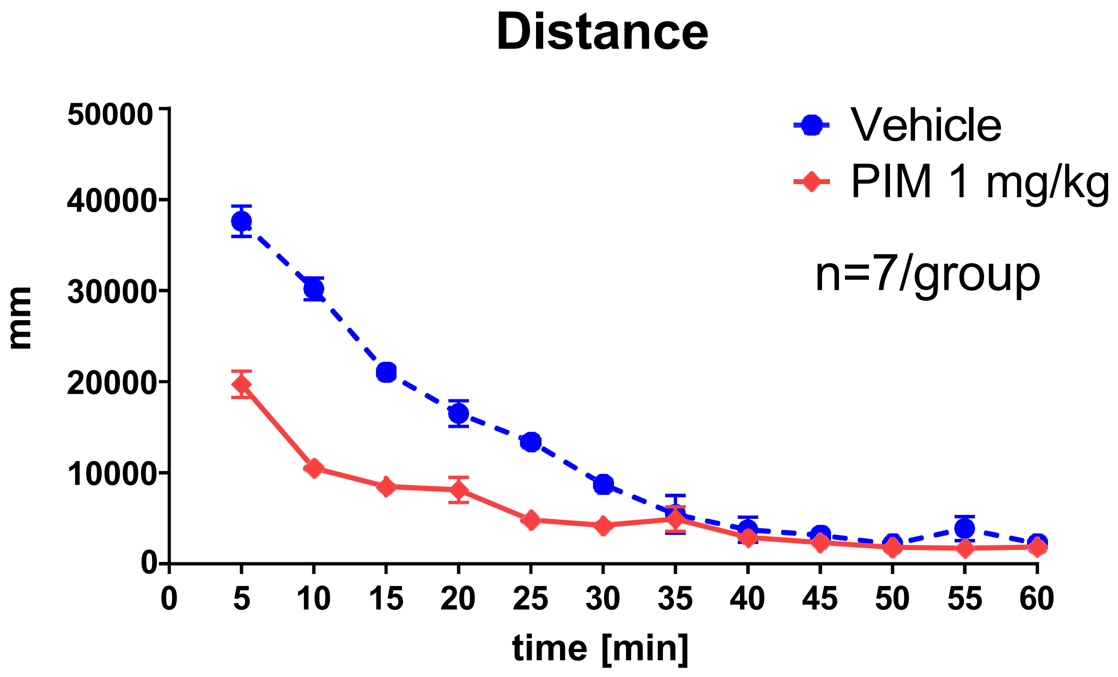

TETRABENAZINE 2 mg/kg, 1x

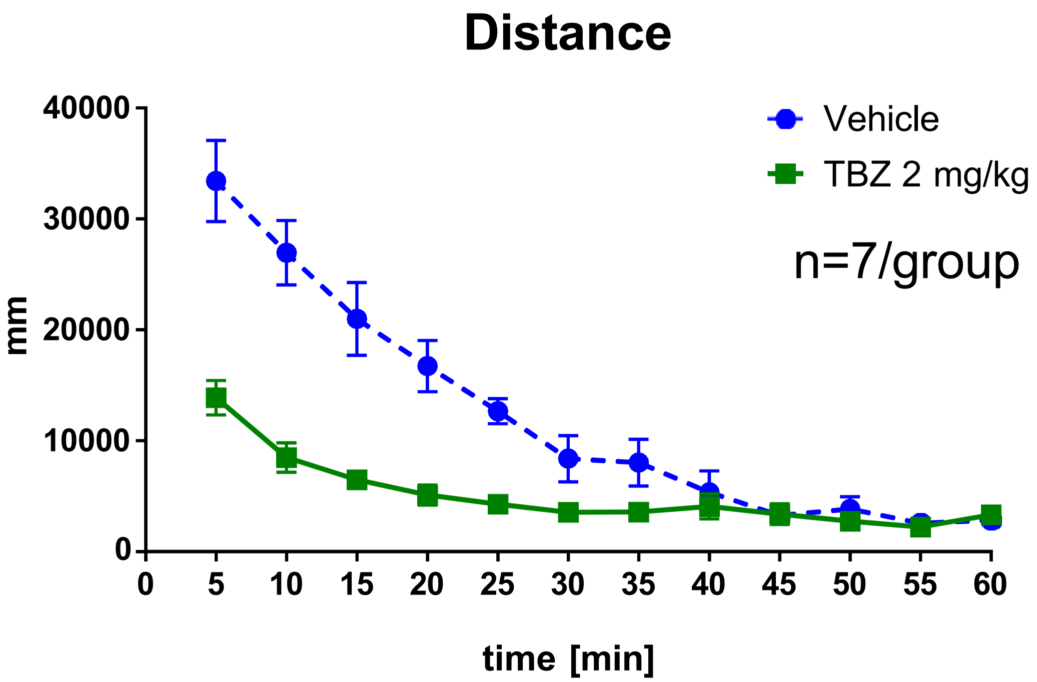

Barbara Kosmowska and Jadwiga Wardas
